# Variations in Alveolar Partial Pressure for Carbon Dioxide and Oxygen Have Additive Not Synergistic Acute Effects on Human Pulmonary Vasoconstriction

**DOI:** 10.1371/journal.pone.0067886

**Published:** 2013-07-31

**Authors:** Quentin P. P. Croft, Federico Formenti, Nick P. Talbot, Daniel Lunn, Peter A. Robbins, Keith L. Dorrington

**Affiliations:** 1 Department of Physiology, Anatomy and Genetics, University of Oxford, Oxford, United Kingdom; 2 Department of Statistics, University of Oxford, Oxford, United Kingdom; Indiana University, United States of America

## Abstract

The human pulmonary vasculature constricts in response to hypercapnia and hypoxia, with important consequences for homeostasis and adaptation. One function of these responses is to direct blood flow away from poorly-ventilated regions of the lung. In humans it is not known whether the stimuli of hypercapnia and hypoxia constrict the pulmonary blood vessels independently of each other or whether they act synergistically, such that the combination of hypercapnia and hypoxia is more effective than the sum of the responses to each stimulus on its own. We independently controlled the alveolar partial pressures of carbon dioxide (Pa_co__2_) and oxygen (Pa_o__2_) to examine their possible interaction on human pulmonary vasoconstriction. Nine volunteers each experienced sixteen possible combinations of four levels of Pa_co__2_ (+6, +1, −4 and −9 mmHg, relative to baseline) with four levels of Pa_o__2_ (175, 100, 75 and 50 mmHg). During each of these sixteen protocols Doppler echocardiography was used to evaluate cardiac output and systolic tricuspid pressure gradient, an index of pulmonary vasoconstriction. The degree of constriction varied linearly with both Pa_co__2_ and the calculated haemoglobin oxygen desaturation (1-So_2_). Mixed effects modelling delivered coefficients defining the interdependence of cardiac output, systolic tricuspid pressure gradient, ventilation, Pa_co__2_ and So_2_. No interaction was observed in the effects on pulmonary vasoconstriction of carbon dioxide and oxygen (p>0.64). Direct effects of the alveolar gases on systolic tricuspid pressure gradient greatly exceeded indirect effects arising from concurrent changes in cardiac output.

## Introduction

The human pulmonary vasculature constricts in response to both hypercapnia and hypoxia [1]–[4]. Sometimes, variations in CO_2_ and O_2_ are such as to work in synchrony on the vasculature. For example, this occurs in a poorly ventilated region of the lung where they both act to direct blood flow away from the region to better ventilated lung tissue, thereby enhancing the efficiency of gas exchange [5]. At other times, variations in CO_2_ and O_2_ are such as to act in opposition on the vasculature. An example is human exposure to high altitude, where the whole lung is exposed to coexisting hypoxia and hypocapnia [6], and the potentially harmful pressor effect of the alveolar hypoxia is obtunded by the dilatory effect of the alveolar hypocapnia. It is not known in what way a combination of the stimuli of hypercapnia and hypoxia affect the blood vessels in the human lung. It is unclear, therefore, whether the effects of the stimuli are additive or synergistic, that is to say, whether variations in O_2_ could potentially enhance the response to CO_2_ or vice-versa.

The question of whether there is a synergy between the effects CO_2_ and O_2_ in the sensing mechanisms of the pulmonary vasculature is of broader interest than in the context of this tissue alone. In relation to the mammalian carotid body a stimulus interaction in the responses of single afferent fibres to CO_2_ and O_2_ has been known since 1975 [7], and considerable attention has been directed at establishing at what cellular level of transduction this synergy might occur [8], [9]. The important consequences of this stimulus interaction on the control of breathing in humans in a wide variety of conditions has been recognized for many years [10], [11]. In comparison, responses of pulmonary vascular smooth muscle to the combined stimuli CO_2_ and O_2_ have received little attention, but are arguably of a similar importance for understanding the behaviour of the lung in health and disease [12], [13].

Animal preparations have not provided a clear indication of what one might expect for the human lung. Most, but not all [14], preparations show vasomotor responses to both respiratory gases, with some degree of synergistic interaction between the effects of CO_2_ and O_2_ being common but variable [15]–[20]. Study of vasoconstrictor responses in the *in vivo* healthy human lung is made particularly difficult by the fact that changes in Pa_co__2_ and Pa_o__2_ induce changes in pulmonary artery pressure and pulmonary vascular resistance (PVR) that are a summation of a *direct* active effect of the gases on vascular smooth muscle and an *indirect* passive effect of concurrent changes in pulmonary blood flow and, potentially, ventilation [21]. The indirect effect may be quite small, because pulmonary vessels tend to be quite distensible, and thus accommodate large changes in flow with little rise in perfusion pressure and with a fall in resistance. This nevertheless makes it misleading to measure either pulmonary artery pressure or PVR as a sole index of pulmonary vascular smooth muscle constriction.

The luxury available in animal preparations of being able to impose a constant pulmonary flow, and using pulmonary artery pressure or PVR as the index of vasoconstriction, has not been achieved in humans [22]. We address this problem by using mixed effects modelling to extract coefficients in direct and indirect pathways linking Pa_co__2_ and Pa_o__2_ with pulmonary artery pressure, and the relative contribution of each pathway. Direct effects of alveolar gases on pulmonary artery pressure are found to dominate. This approach also evaluates whether the gases have an additive or synergistic action; an additive action is observed, consistent with the approach adopted in an earlier model of feedback control of regional gas exchange in the human lung [13].

## Methods

### Ethics Statement

The study was approved by the Oxfordshire Research Ethics Committee and performed in accordance with the Declaration of Helsinki. Informed written consent was obtained from all volunteers.

### General approach to the measurement of pulmonary vasoconstriction

The general approach adopted was to use non-invasive measurement of systolic pulmonary artery pressure as our index of pulmonary vasoconstriction, whilst at the same time taking into account the dependence of this pressure upon other variables: ventilation and cardiac output. This separation of *direct* and *indirect* influences of Pa_co__2_ and Pa_o__2_ on systolic pulmonary artery pressure was achieved using mixed effects modelling.

### Volunteers

Nine healthy volunteers (5 women and 4 men), aged 24±4 years and with BMI 22.5±2 kg/m^2^ (mean ± S.D.), completed the study. Female volunteers were asked to participate only during the first 14 days of their menstrual cycle. Volunteers visited the laboratory before undergoing the experimental protocols in order to discuss the procedures and confirm that they were suitable for echocardiographic assessment of tricuspid regurgitation.

### Study design

The pulmonary vascular response to four different levels of P_co2_ was studied at each of four different levels of P_o2_. This led to 16 different combinations of P_co2_ and P_o2_ overall, each called a protocol. Each protocol comprised a ten-minute exposure to the particular P_co2_/P_o2_ combination which was preceded by 5 min of baseline conditions (see below). Cardiovascular and respiratory variables were measured throughout each protocol.

Each volunteer completed the sixteen protocols in one of four different orders, determined by block randomization based on date of first contact. Volunteers completed these protocols in two batches of eight in two afternoons. Each protocol was preceded by at least ten minutes of quiet rest. The sixteen protocols were the sixteen combinations of four levels each of end-tidal partial pressures of CO_2_ (Pet_co__2_) and O_2_ (Pet_o__2_). These end-tidal values were assumed to be equivalent to alveolar partial pressures. The following four levels of Pet_co__2_ were chosen (relative to normal baseline): +6, +1, −4 and −9 mmHg. The levels of Pet_o__2_ used were 175, 100, 75 and 50 mmHg. This provided an opportunity to span the range from relative hyperoxia to the hypoxia used in other studies [23]–[25], and so cover the likely regional values for these variables encountered within the healthy lung at sea level [13], [26].

### Gas control

Pet_co__2_ and Pet_o__2_ were controlled using an end-tidal forcing system as previously described [27]–[29]. Volunteers lay in a semi-left lateral position and breathed through a mouthpiece with the nose occluded. Ventilatory volumes and flows were measured by turbine and pneumotachograph respectively. Gases were sampled by a catheter close to the mouth and analysed continuously by mass spectrometry.

Ventilation during the protocols conducted at Pet_co__2_ values of −9 and −4 mmHg was achieved by voluntary hyperventilation. Volunteers controlled the frequency of breathing through the use of an audible metronome, and the depth of breathing through feedback presented on an oscilloscope connected to the output of the turbine measuring ventilatory flows. Ventilation during the protocols conducted at Pet_co__2_ values of +6 and +1 mmHg was spontaneous. Each protocol consisted of 5 min of spontaneous ventilation, or voluntary hyperventilation, with end-tidal gases held constant at baseline values (100 mmHg Pet_o__2_ and the measured baseline Pet_co__2_) followed by ten minutes with these gases at the specified levels for the protocol. For protocols involving hypocapnia, a constant combination of breathing depth and frequency was used throughout.

### Echocardiography

In approximately 70% of healthy volunteers it is possible to detect with Doppler ultrasound a regurgitant blood flow from the right ventricle to the right atrium during ventricular systole. Measurement of the peak velocity (v) of this regurgitant jet affords an opportunity to estimate the systolic pressure difference ΔPmax between the right ventricle (where the pressure is close to pulmonary artery systolic pressure) and right atrial pressure. This relationship is given by the Bernoulli equation: ΔPmax = ρv^2^/2, where ρ is blood density. The peak systolic tricuspid pressure gradient (ΔPmax) and cardiac output were measured using a GE Vivid-i ultrasound machine with a S4 transducer (2–4 MHz). Assessment of ΔPmax used Doppler echocardiography, via a 4-chamber view of the heart, to measure the peak pressure difference between the right ventricle and the right atrium during systole. Since right atrial pressure changes little during hypoxia, changes in ΔPmax reflect changes in systolic pulmonary arterial pressure [30], [31]. The utility of measuring ΔPmax as an index of pulmonary vascular constriction in healthy humans has been shown during hypoxia [24], [25], hypercapnia and hypocapnia [2], [13].

Cardiac output (Q̇) was measured using Doppler echocardiography to assess non-turbulent flow through the centre of the left ventricular outflow tract (LVOT). The cross-sectional area of the LVOT was obtained by measuring the diameter of the aortic valve using a parasternal long-axis view of the heart. Flow through the LVOT was imaged using an apical five-chamber view of the heart and measured using the velocity-time integral. Systolic flow was multiplied by the cross-sectional area of the LVOT to provide an estimate of stroke volume. Heart rate was recorded simultaneously. The stroke volume was multiplied by the heart rate to provide an estimate of cardiac output.

For both measurements, results depend to some extent upon the phase of the respiratory cycle, so end-expiration was chosen as the phase of that cycle giving minimal disturbance; images of the spectral traces at or as near as possible to end-expiration were saved digitally for later analysis.

### Data analysis

Ventilation (V̇e) and end-tidal gases were assessed using 30 s averages of the values calculated from each breath. For ΔPmax and Q̇, approximately five measurements of each variable were obtained each minute and then 2 min averages were calculated.

Baseline variables were the average of values recorded during the first five minutes of each protocol. Protocol variables were the average of the last six minutes of each protocol. The change in each variable was the difference between the protocol and baseline values.

Pet_o__2_ values were converted to an equivalent fractional oxyhaemoglobin saturation (So_2_) using the equation provided by Severinghaus [32]. Although the major stimulus to pulmonary vascular constriction is the partial pressure of the sensed gases, the response to oxygen is known to be markedly non-linear and the purpose of this sigmoid transformation was to permit us to use a virtual saturation in place of P_o2_ in our analysis, and thereby assess the suggestion of previous authors [33] that hypoxic constriction tends to be a linear function of So_2_ whilst being a markedly curvilinear function of P_o2_.

### Modelling and statistical analysis

The experimental data were analysed using the following linear model:
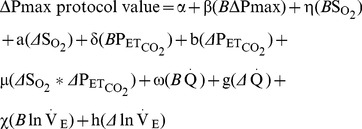
(1)where *B*ΔPmax, *B*So_2_, *B*Pet_co__2_, *B*Q̇ and *B*lnV̇e refer to baseline values of the respective variables ΔPmax, So_2_, Pet_co__2_, Q̇ and lnV̇e, whilst *Δ*So_2_, *Δ*Pet_co__2_, *Δ*Q̇ and *Δ*lnV̇e refer to the differences between protocol and baseline values. *Δ*So_2_*ΔPet_co__2_ allows for possible interaction between the stimuli. The logarithm of V̇e was required in the analysis instead of V̇e itself so as to avoid giving undue dominance to a small number of high values of V̇e. The coefficients preceding each term were obtained by fitting the model to the experimental data.

**Figure 1:**
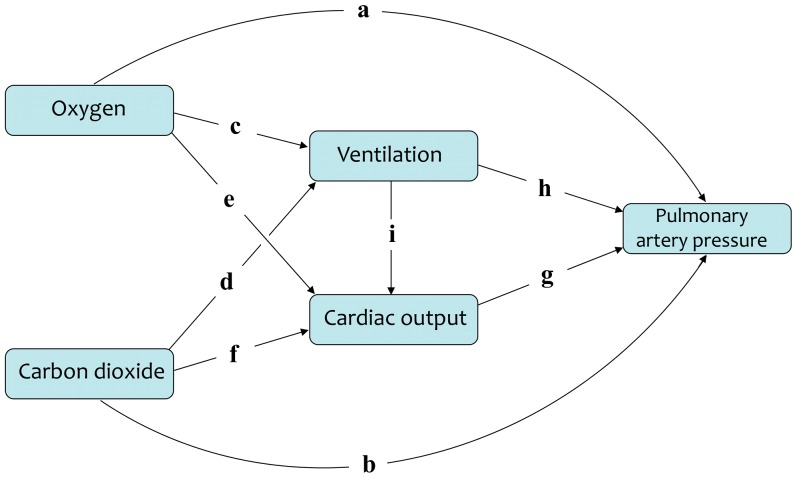
Diagram of the relationships involved in the study. ΔPmax is viewed as the primary measure of pulmonary vasoconstriction, influenced *directly* by alveolar gases (pathways a and b), whilst also being a weak function of cardiac output and possibly ventilation (pathways g and h). The latter two are also functions of alveolar gases (via the pathways c–f). Interactions are not represented.

Figure 1 shows the conceptual framework for our modelling approach. ΔPmax is viewed as primarily a measure of pulmonary vasoconstriction dependent upon a *direct* effect of alveolar gases on vascular smooth muscle, whilst also being a weak function of Q̇ and V̇e. These in turn are functions of alveolar gases, and provide an indirect route via which alveolar gases can change ΔPmax. The modelling described below delivers mean values plus confidence intervals, expressed as standard error of these means, to the nine coefficients displayed in Figure 1, as well as assessing the significance of the interactive term *Δ*So_2_*ΔPet_co__2_ in *Eq. 1*.

The data were analysed with linear mixed effects modelling to account for correlation within individual volunteers and for variability between volunteers. A two-level multilevel model with an exchangeable correlation structure was fitted. This statistical technique can be used for analysing data that occur as repeated measurements on each of a number of participants in order to identify and quantify responses common to all participants, taking into account individual variability, with no two individuals being the same. Models similar to that in *Eq. 1* were derived for Q̇ and ln(V̇e).

Data were analysed using ‘R’, open-source computer software for statistical analyses. R uses a penalised likelihood method to fit the data to a given model iteratively until no improvement in the residual deviance is achieved. Data were initially fitted to a model in which all of the possible contributing factors in *Eq. 1* were considered. The model was then adjusted to exclude the least significant factor until all remaining factors showed significance with p<0.05. This provided individual coefficients for each contributing factor that define the linear relationships. Each coefficient was then fitted as a random variable, with the mean and standard deviation estimated from the data, retaining adjustments that enhanced the explanatory power of the model. This was judged by two methods: first, if the random factor correlated well with another random factor then no additional explanatory power was added, the variability being explicable by one of the two factors. The constant in the model (which provides the y-axis intercept on a graph of the function) was always modelled as a random factor, and if it correlated well with another random factor then it was acting as a surrogate for that factor and the factor could be subsumed by the intercept factor. Secondly, if the residual deviance was not decreased by a large amount then the explanatory power was not enhanced, and the addition of a random factor was not necessary.

## Results

**Figure 2:**
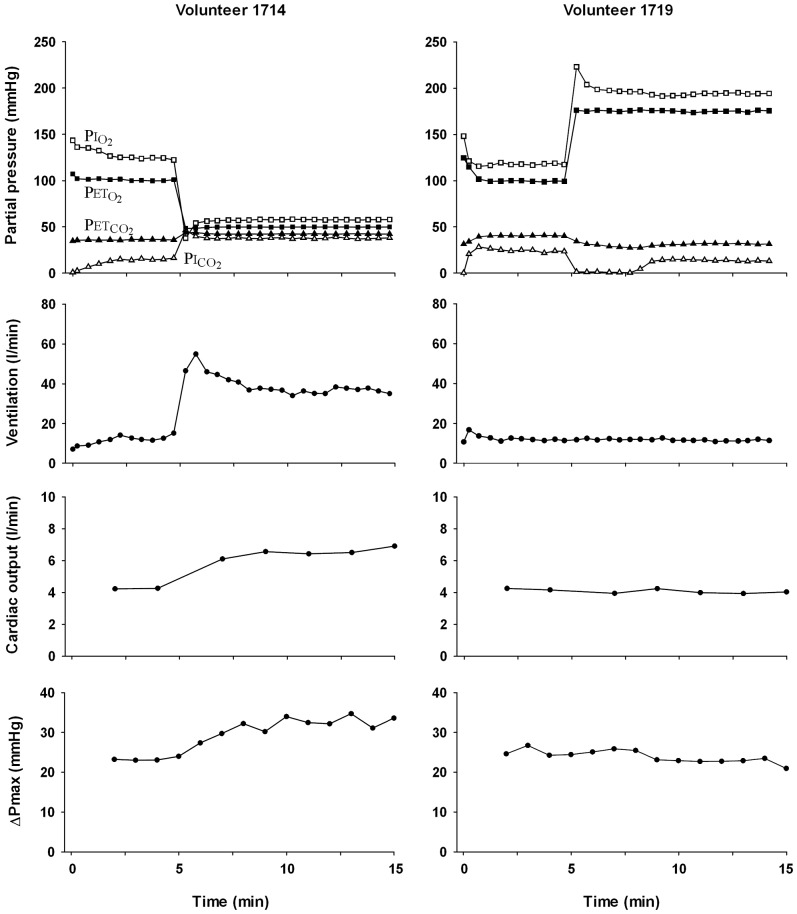
Example data from two protocols on different volunteers. In each protocol the end-tidal gases were held at normal euoxic and eucapnic values for the baseline period of 5 min and then stepped to individual target protocol values. These were as follows: left panels: volunteer 1714 with hypercapnia (Pet_co__2_ = baseline+6 mmHg) and hypoxia (Pet_o__2_ = 50 mmHg) using spontaneous hyperventilation; right panels: volunteer 1719 with hypocapnia (Pet_co__2_ = baseline−9 mmHg) and hyperoxia (Pet_o__2_ = 175 mmHg) using voluntarily controlled constant hyperventilation. Upper panels: inspired oxygen (Pi_o__2_) and carbon dioxide (Pi_co__2_) partial pressures and end-tidal oxygen (Pet_o__2_) and carbon dioxide (Pet_o__2_) partial pressures. Middle panels: ventilation and cardiac output. Lower panels: ΔPmax. Respiratory data represent means of multiple measurements (one per breath) in each time period.

Protocols were conducted between August 2007 and June 2009. Figure 2 shows representative data from two protocols that illustrate spontaneous ventilation during hypercapnia and controlled ventilation to induce hypocapnia. The left panel shows a protocol involving hypoxia with hypercapnia, and the right panel shows a protocol involving hypocapnia with hyperoxia. The upper panels show the control of Pet_o__2_ and Pet_co__2_ for the two protocols; gas control achieved a rapid (<1 min) step from euoxia and eucapnia to protocol values and little variation from target end-tidal values either side of the change. The middle panels show the ventilations and cardiac outputs achieved during the protocols and the bottom panels show the values of ΔPmax recorded during the protocols. Table 1 gives the accuracy to which the gas control was achieved for each of the four levels of Pet_co__2_ and the four levels of Pet_o__2_ that were targeted in the protocols. It can be seen that for CO_2_ the errors in gas control are well below 0.1 mmHg, whilst for O_2_ the errors in gas control are around 1 mmHg. Table 2 gives the individual changes in ΔPmax for each of the sixteen protocols.

**Table 1:** Errors (mean and standard deviation) in control of end-tidal gases calculated as the measured end-tidal partial pressure minus the target end-tidal partial pressure for the four levels of CO_2_ and four levels of O_2_ used in the study.

	CO_2_ error (mmHg)			
Target PCO_2_ (mmHg)	−9	−4	1	6
Error	0.023	−0.008	0.018	−0.051
SD	0.325	0.256	0.255	0.352

	O_2_ error (mmHg)			
Target PO_2_ (mmHg)	175	100	75	50
Error	−1.315	−1.201	0.610	0.813
SD	1.792	0.766	1.100	1.557

**Table 2:** Individual changes in systolic tricuspid pressure gradient (ΔPmax) in response to sixteen combinations of end-tidal gas composition.

	Change in ΔPmax (mmHg)															
End-tidal PO_2_ (mmHg)	50 mmHg				75 mmHg				100 mmHg				175 mmHg			
Change in end-tidal PCO_2_ (mmHg)	+6	+1	−4	−9	+6	+1	−4	−9	+6	+1	−4	−9	+6	+1	−4	−9
**Subject 1662**	12.7	10.4	3.1	2.5	10.6	3.7	2.0	0.8	2.6	1.4	1.0	−1.2	5.6	0.1	0.1	−0.5
**Subject 1664**	3.9	3.5	3.7	3.4	0.0	0.0	0.0	0.1	3.0	1.9	0.3	−1.7	2.4	1.3	−0.6	−0.7
**Subject 1701**	6.7	6.1	5.1	2.5	3.6	0.9	0.6	1.1	3.0	1.3	−0.5	−0.8	1.7	−0.2	−0.2	0.4
**Subject 1703**	5.9	1.6	3.7	1.9	5.5	2.0	0.0	1.5	2.8	1.9	2.5	1.0	3.4	0.5	0.2	2.1
**Subject 1714**	9.7	10.7	6.6	7.3	7.8	1.8	2.3	−0.8	5.8	0.1	−0.2	0.6	2.4	−0.7	−0.4	0.1
**Subject 1719**	15.1	12.6	15.0	13.8	4.7	3.6	5.2	−1.6	3.9	0.0	−0.7	0.8	3.2	0.0	1.4	−2.4
**Subject 1730**	4.2	6.0	3.8	2.0	2.9	1.1	−1.7	2.2	3.6	1.2	1.0	1.1	0.8	−0.7	−2.6	−1.8
**Subject 1096**	12.4	12.7	9.9	7.2	2.6	0.8	−1.8	−0.7	3.2	−1.0	−0.2	−1.9	1.0	−1.5	−2.5	−0.9
**Subject 1751**	6.8	7.9	4.7	5.4	1.2	−0.1	1.5	1.3	−0.7	0.6	1.5	−0.8	0.1	−0.1	−0.7	−1.3
**Mean**	**8.6**	**7.9**	**6.2**	**5.1**	**4.3**	**1.6**	**0.9**	**0.4**	**3.0**	**0.8**	**0.5**	**−0.3**	**2.3**	**−0.1**	**−0.6**	**−0.6**
**SEM**	**1.3**	**1.3**	**1.3**	**1.3**	**1.1**	**0.5**	**0.7**	**0.4**	**0.6**	**0.3**	**0.4**	**0.4**	**0.6**	**0.3**	**0.4**	**0.4**

Volunteers were exposed to each combination of end-tidal P_O_2__ and P_CO_2__ for 10 min, preceded by 5 min baseline breathing with end-tidal gases held close to baseline values (100 mmHg end-tidal P_O_2__ and the measured baseline end-tidal P_CO_2__). The change in peak systolic tricuspid pressure gradient (ΔPmax) was calculated as the difference between the mean baseline ΔPmax and the mean ΔPmax during the last 6 minutes of each protocol. Gas control was achieved by means of end-tidal forcing.

### Results of statistical analysis

A major objective of this study was to investigate whether the stimuli of hypercapnia and hypoxia constrict the pulmonary blood vessels independently of each other, or whether they act synergistically; in other words, evidence of an interaction *Δ*So_2_*ΔPet_co__2_ was sought.

The main analysis used the model given in *Eq. 1*. Of the included factors baseline Q̇, baseline Pet_co__2_, baseline V̇e, baseline So_2_ and *Δ*So_2_*ΔPet_co__2_ were all removed from the model sequentially, in that order, without significantly worsening the fit, suggesting that they had no significant role in determining ΔPmax protocol value. The interactive term was insignificant at the level p>0.64.

To ensure the study had sufficient power to detect any interaction between the effects of hypoxia and hypercapnia, we calculated power as a function of the percentage change of the ΔPmax response attributable to the interaction term (*Δ*So_2_*ΔPet_co__2_). At the 5% significance level, the study had a power of 80% for the detection of a 4% change in the ΔPmax response due to interaction; the power for detecting a 10% change in the response was close to 100%. Despite adequate power, no evidence of an interaction was identified.

The final model fitted the following equation:
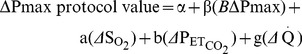
(2)where the coefficients are given in Table 3 as a value ± standard error. The model that best explains the experimental data delivers coefficients β and b as fixed coefficients with α, a and g as coefficients that vary between individuals with normal distributions and standard deviations of 0.59 mmHg, 0.26 mmHg/%desaturation and 0.36 mmHg/l/min, respectively.

**Table 3:** Model coefficients for interdependence of systolic tricuspid pressure gradient (ΔPmax), cardiac output (Q̇), ventilation (expressed as the natural logarithm of ventilation, ln(V̇e)), end-tidal partial pressure of CO_2_ (Pet_co__2_) and end-tidal oxygen level expressed as equivalent haemoglobin saturation (So_2_).

Protocol value	Intercept	Baseline	ΔSo_2_	ΔPet_co__2_	ΔQ̇	ΔlnV̇e
ΔPmax	α	β	a	b	g	h
	3.4±1.5	0.89±0.06	0.43±0.09	0.18±0.03	0.66±0.32	0
	mmHg		mmHg/%desat		mmHg/l/min	mmHg/ln(l/min)
Q̇	γ	ε	e	f		i
	1.1±0.3	0.79±0.06	0.06±0.01	0.02±0.01		0.33±0.14
	l/min		l/min/%desat	l/min/mmHg		l/min/ln(l/min)
ln(V̇e)	λ	ζ	c	d		
	1.2 ±0.2	0.52±0.08	0.039±0.004	0.099±0.009		
	ln(l/min)		ln(l/min)/%desat	ln(l/min)/mmHg		

Roman alphabet coefficients are depicted in Fig. 1. Roman and Greek coefficients are defined in Eqs. 2, 3 and 4. Coefficients are given as a value ± standard error.

**Figure 3:**
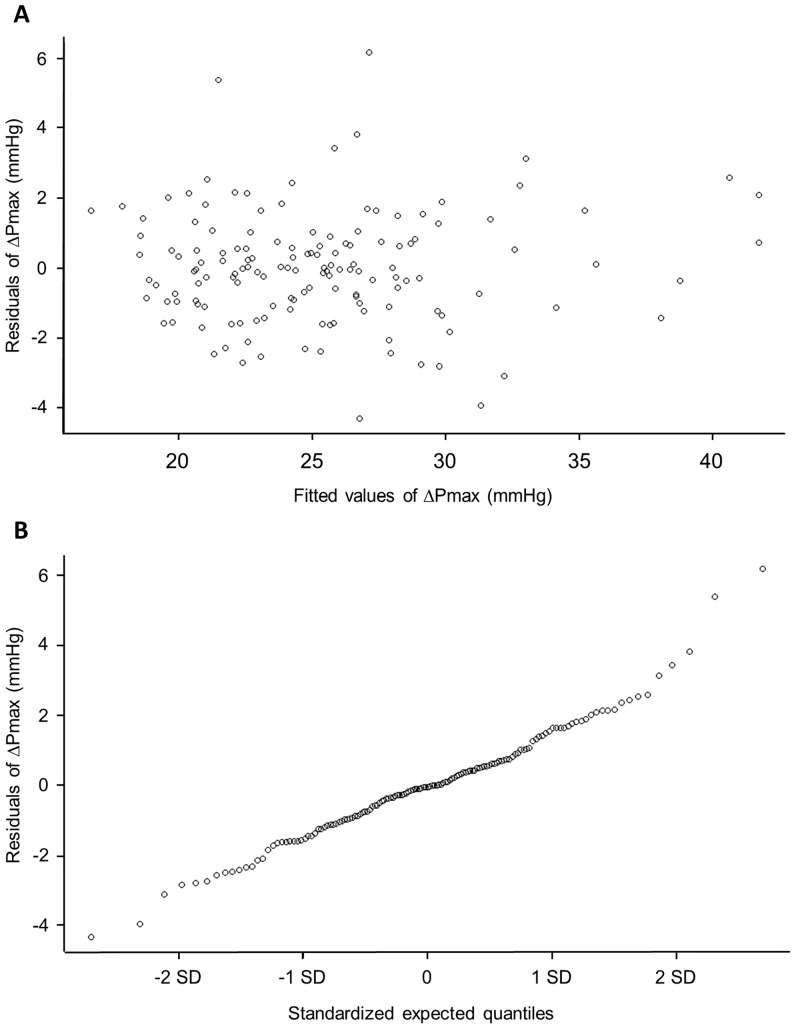
Plots of residuals for ΔPmax associated with model in *Eq. 1*. (A) Residuals for ΔPmax plotted against the values for ΔPmax fitted to the model in *Eq. 1*. A skewed plot would show that the assumption of constant variance had been violated. No such pattern is discernible in this plot. (B) Residuals for ΔPmax plotted against the standardized expected quantiles (units of standard deviation) fitted to the model in *Eq. 1*. The linear relationship demonstrates that the residual deviances map on to a Normal distribution.

The usual linear regression assumptions of normality and constant variance are confirmed by plotting the residuals against the fitted values (Fig. 3A) and inspection of a normal residuals-quantile plot (Fig. 3B). The purpose of the former plot is to show whether variance changes throughout the range of data, which would appear as a trend for the residuals to deviate from 0 as a function of the fitted values. One or two outliers on a dataset of this size are to be expected and are not necessarily inconsistent with a good fit. The latter plot shows whether the data are approximately normal, an assumption which is violated to the extent that the plot deviates from being linear.

The independent effects of altered Pet_co__2_ and So_2_ on Q̇ were modelled using the same approach. The analysis fitted the equation:
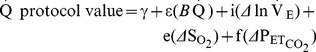
(3)where the coefficients are given in Table 3. The model delivered ε, i, e and f as fixed coefficients, whilst γ was taken to be normally distributed with a standard deviation of 0.08 l/min.

A similar approach was used for lnV̇e. Data from protocols involving hypocapnia were excluded from this analysis because V̇e was consciously controlled in these protocols in order to achieve hypocapnia. The final model for V̇e derived the following equation:
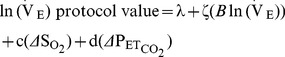
(4)where the coefficients are given in Table 3. The model delivered ζ, c and d as fixed coefficients, whilst λ was taken to be normally distributed with a standard deviation of 0.22 ln(l/min).

**Figure 4:**
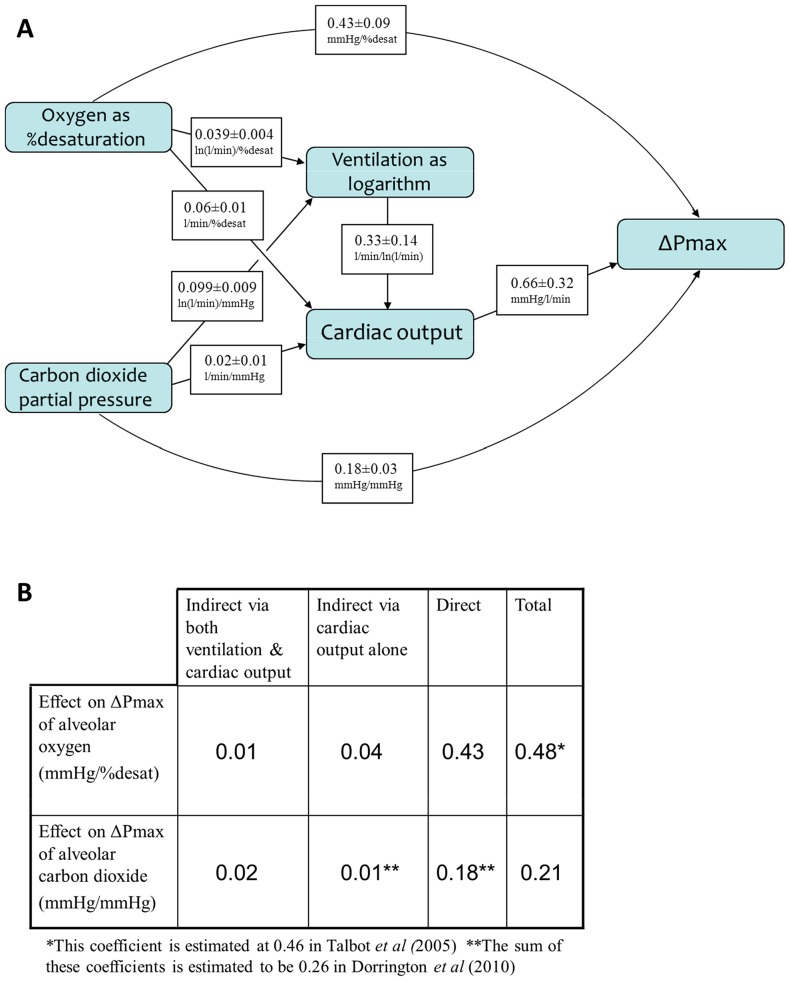
Coefficients obtained from modelling studies. (A) Results for the coefficients from Fig. 1 obtained by mixed effects modelling, given as mean ± standard deviation. (B) Components of direct and indirect pathways whereby alveolar oxygen and carbon dioxide influence ΔPmax, with comparisons with earlier studies [13], [40]. For both gases the direct pathway dominates.

Figure 4 gives the results for the coefficients defined in Fig. 1, and summarizes direct and indirect pathways via which O_2_ and CO_2_ influence ΔPmax. For both gases, the direct pathway dominates.

## Discussion

The main finding of this study is that the effects of CO_2_ and O_2_ on human pulmonary artery pressure are additive rather than synergistic. Specifically, the retention in the model for systolic pulmonary artery pressure of a term incorporating the product of oxyhaemoglobin saturation and carbon dioxide partial pressure could not improve the predictive power of the model. An additional finding is that the direct effects of alveolar gases on pulmonary artery pressure via vasoconstriction dominate the indirect effects that come about via changes in ventilation and cardiac output.

Methods for measuring pulmonary vasoconstriction *in vivo* are controversial. In reduced preparations, typically perfusions of non-human animal lungs or vessels *in vitro*, it is common to manipulate pulmonary flow to be constant and then use either the pressure drop across the pulmonary circulation or PVR as measures of vascular ‘tone’ or ‘constriction’ [34], [35]. An alternative approach is to maintain perfusion pressure constant, and associate changes in vascular constriction with changes in blood flow [20], [36]. In awake humans neither of these approaches has proved accessible, and measurements of pulmonary vasoconstriction are complicated by the fact that both pulmonary arterial pressure and pulmonary blood flow usually change in response to changes in alveolar gases. A common invasive strategy has been to measure PVR using a Swan-Ganz pulmonary artery catheter, whilst accepting that changes in PVR occur independently in response to changes in both cardiac output [37] and alveolar gas composition [38]. This study demonstrates that the non-invasive measurement of systolic pulmonary artery pressure using Doppler ultrasound is a useful tool to assess vasoconstriction in response to changes in alveolar gases, as long as account is taken, as with catheter measurements, of the separate effect of cardiac output on this variable.

### Comparison of pulmonary vascular response with previous human studies

Fig. 4(B) suggests for this study that 10–15% of the effect of alveolar gases on ΔPmax occurs via indirect pathways. Two such pathways have been identified here: changes in cardiac output induced by changes in ventilation alone, and changes in cardiac output induced by CO_2_ and O_2_ in the absence of changes in ventilation. Few data are available from the literature for comparison. A study focusing on longer durations of hypoxia (0.5–8 h) found that approximately 5% of the rise in ΔPmax with hypoxia could be attributed to *indirect* effects via cardiac output [39].

The sensitivity of ΔPmax to acute changes in Q̇ is defined by coefficient g in *Eq. 2* and Fig. 1. The contribution of Q̇ alone is defined by g = 0.66 mmHg/l/min. A previous study [39] observed spontaneous concurrent changes in ΔPmax with changes in Q̇ during air breathing in the absence of changes in alveolar gas composition and found a value for g of 0.60 mmHg/l/min, in good agreement with that found here.

Other coefficients accessible from previous studies on similar human volunteers permit estimates for e (0.06 l/min/%desat from hypoxic exposures [13], [40]; here identically 0.06 l/min/%desat) and f (0.04 l/min/mmHg from hypocapnic exposures at constant ventilation, [13]; here 0.02 l/min/mmHg).

### Limitations of the study

The study measured changes in cardiopulmonary variables between 4 and 10 min after induction of new values of alveolar gases. A maximum exposure of 10 min to the perturbation in alveolar gas composition was chosen in part because of the difficulty experienced by volunteers in tolerating longer exposure to extremes such as combined hypoxia (Pet_o__2_ = 50 mmHg) and hypercapnia (Pet_co__2_ = +6 mmHg). Previous work has suggested that this is a sufficiently long period in which to capture the initial acute phase of human hypoxic pulmonary vasoconstriction and the hypoxic increase in cardiac output, in which the time constants of the responses are around 2 min [40], [41]. Recent work has found, however, that the time courses of the acute human cardiopulmonary responses to euoxic hypercapnia and hypocapnia have time constants in the range 4–10 min [13], suggesting that the present experiments have measured a substantial but partial component of the acute changes in ΔPmax and Q̇ to changes in Pa_co__2_. It is consequently difficult to obtain reliable estimates from previous studies for comparison with coefficients b and f in *Eqs. 2* & *3*; this may be why it is these coefficients that agree least well with estimates from previous studies. With regard to the coefficients relating to the cardiopulmonary responses to oxygen, values for a, g, & e show fair agreement with published values obtained from well-defined steady-state measurements.

A second limitation of the study arises from the requirement to establish voluntarily controlled ventilation for half of all measurements made, in order to achieve hypocapnia. The resulting halving of the number of data pertaining to the coefficients linking to ventilation in Fig. 1 will have reduced the precision with which coefficient i in *Eq. 3* could be estimated and reduced the probability of detecting a small but non-zero value of the coefficient h linking ΔPmax directly with V̇e.

Thirdly, this study did not seek to understand the cellular basis for any interaction between CO_2_ and O_2_ in the pulmonary circulation, but instead to understand the effects of alveolar gas composition at the integrative level in humans. For example, we did not address the question of whether changes in pulmonary vascular tone result directly from alterations in P_CO_2__, or whether they are secondary to the associated change in pH. This question has been addressed in animal studies, some of which suggest an effect of hypercapnia *per se* in the pulmonary vasculature [42], [43], but further studies would be needed to explore this issue in humans.

### Physiological significance of the findings

An accurate appreciation of the way in which the stimuli CO_2_ and O_2_ work together on pulmonary vessels is of importance to the understanding of situations in which they act in synchrony or in opposition. The spontaneous matching of perfusion to ventilation in the lung is usually modelled as being achieved solely by the vasoconstrictor effects of hypoxia on small pulmonary arteries [44], [45], but the local vasoconstrictor effect of hypercapnia has the potential to enhance this matching [1], [36]. It remains a possibility that the effects of hypoxia and hypercapnia acting only within an isolated small region of lung tissue might display a different, possibly interactive, relationship from the global effects on all lung tissue studied here. One possible reason for this is that the experiments subjected volunteers to relatively stressful perturbations in end-tidal gas composition that might lead to global autonomic effects on the pulmonary circulation that would not occur with perturbations limited to small regions of lung tissue. Even on the assumption of additive, rather than interactive, effects of the two stimuli recent calculations suggest that CO_2_ may play a more substantial role than O_2_ in ventilation-perfusion matching in the healthy lung at sea level [13]. Under conditions of therapeutic artificial ventilation, clinicians recognize the potential adverse effect on oxygenation of the patient of a low Pa_co__2_ in a hyperventilated hypoxic lung leading to inhibition or elimination of hypoxic vasoconstriction in that lung [46], [47], but the relative contributions of the stimuli have remained unclear.

Pulmonary hypertension at high altitude is associated with global hypoxia with hypocapnia throughout the lung [10] and appears to be responsible for high altitude pulmonary edema in patients who have an exaggerated vasoconstrictor response [48]. It remains uncertain to what extent in affected individuals a weak vasodilatory effect of hypocapnia might inadequately ameliorate the pulmonary hypertension that results from a strong vasoconstrictor effect of hypoxia, because these stimuli have not been examined separately in this setting [49]. The human lung shows considerable potential to dilate in response to sustained hypocapnia [2], and it would clearly be beneficial at altitude for there to be a balance between the vasodilatory effects of hypocapnia and the constriction brought about by hypoxia. The present experiments have quantified the extent of this balance for very acute responses in the period 4–10 min following a step change of alveolar gases. Further work is required to find whether the considerably more intense responses to more sustained combinations of CO_2_ and O_2_ stimuli, such as those occurring over hours and days at high altitude, combine in a similar additive manner.

A novel finding from the study has been the possibility of obtaining a quantitative estimate of the effect of V̇e on Q̇ that is independent of the effects of alveolar gases, namely the coefficient i. The value of i = 0.33 l/min/ln(l/min) suggests a 0.33 l/min rise in cardiac output attributable to a 2.72-fold rise in ventilation. Another interpretation, assuming linearity over a broad range of ventilation, is that a rise in ventilation from a resting value of about 4.5 l/min to a twenty-fold value of 90 l/min associated with very vigorous exercise might contribute a rise in cardiac output of ∼1 litre/min from the direct effect of ventilation on the cardiovascular system alone. Interestingly, ventilation alone appears to have no direct effect upon ΔPmax (i.e. h = 0). Further studies are required to establish the magnitude of these interrelationships over wider ranges of physiological disturbance.
